# Laparoscopic sacrohysteropexy and myomectomy for uterine prolapse: a case report and review of the literature

**DOI:** 10.1186/1752-1947-3-99

**Published:** 2009-11-03

**Authors:** Radwan Faraj, Jonathan Broome

**Affiliations:** 1 Department of Obstetrics and Gynaecology, Royal Bolton Hospital, Minerva Road, Farnworth, BL4 0JR, UK; 2 Urogynaecology Department, Royal Bolton Hospital, Minerva Road, Farnworth, BL4 0JR, UK

## Abstract

**Introduction:**

A large number of hysterectomies are carried out for uterine prolapse, menorrhagia and other symptomatic but benign gynaecological conditions, which has increased interest in new approaches to treat these disorders. These new procedures are less invasive and offer reduced risk and faster recovery.

**Case presentation:**

Sacrohysteropexy can be carried out instead of vaginal hysterectomy in the treatment of uterine prolapse. It involves using a synthetic mesh to suspend the uterus to the sacrum; this maintains durable anatomic restoration, normal vaginal axis and sexual function. A laparoscopic approach has major advantages over the abdominal route including shorter recovery time and less adhesion formation. We describe a laparoscopic sacrohysteropexy in a 55-year-old Caucasian British woman that was technically difficult. An intramural uterine fibroid was encroaching just above the uterosacral ligament making mesh positioning impossible. This was removed and the procedure completed successfully.

**Conclusion:**

Posterior wall fibroid is not a contraindication for laparoscopic sacrohysteropexy. This procedure has increasingly become an effective treatment of uterine prolapse in women who have no indication for hysterectomy.

## Introduction

Uterine prolapse is the protrusion of the uterus down into, and sometimes through, the vagina. It can affect quality of life by causing symptoms of pressure and discomfort, and by its effects on urinary, bowel and sexual function. Current treatment options include pelvic floor muscle training, use of pessaries and surgery.

This case report discusses uterine suspension using mesh for uterine prolapse and involves attaching the uterus to the sacrum (sacrohysteropexy). This procedure preserves the normal uterus and can be performed via an abdominal or laparoscopic approach. Its efficacy and safety are discussed together with a review of the published literature.

## Case presentation

A 55-year-old Caucasian British woman was referred by her general practitioner with a dragging sensation in her lower pelvis and vagina of 18 months' duration. She described it as 'my womb is going to drop out'. She reported very vague symptoms during micturition and had recurrent cystitis. She did not complain of any bowel symptoms or urinary incontinence, and did not have significant sexual dysfunction.

Two years before her presentation, she experienced urinary urgency with urge incontinence and underwent video urodynamics. This showed signs of an overactive bladder and the anticholinergic, tolterodine extended release (ER) was prescribed, which has improved her symptoms dramatically.

Her obstetric history included three normal deliveries, the last one 27 years previously (birth weight 4-4.5 kg). On examination, there was no obvious cystocoele or stress incontinence seen in the supine position. The cervix looked healthy and on Sims' speculum examination, there was a very mild degree of prolapse of the anterior and posterior vaginal wall. There was an enterocele and the cervix had descended to within the introitus but outside the introitus with strain (stage III modified Baden and Walker).

**Figure 1:**
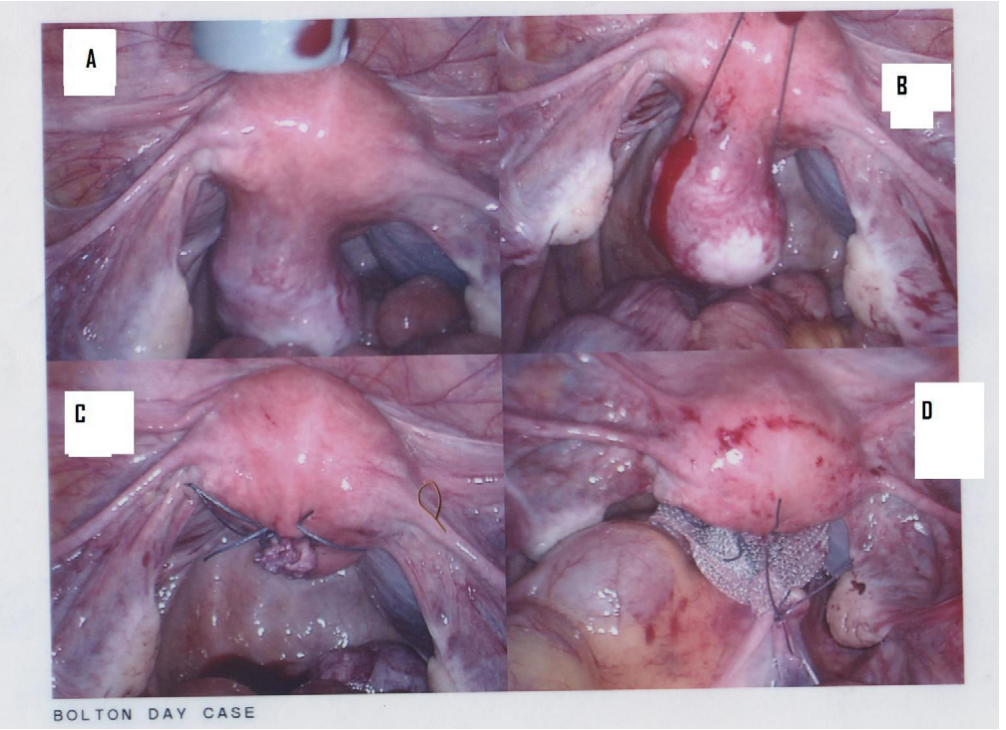
**A. Posterior myoma**. B. Endoloop surrounding the myoma base. C. Myoma enucleated from the posterior uterine wall. D. Mesh application between the posterior uterus at the uterosacral ligaments and the sacral promontory where the peritoneum is resutured.

Considering the primary uterine prolapse and the dragging sensation, she was counselled to undergo laparoscopic sacrohysteropexy. At laparoscopy, she was found to have a moderate sized (6×7 cm) pedunculated fibroid coming off the back of the uterus, which may account for some of her pelvic dragging sensations (Figure 1A).

The fibroid was removed using an endoloop and two sutures (Figures 1B and 1C). Then sacrohysteropexy was performed to further elevate the uterus: polypropylene Ethicon mesh (Espiner Medical) was fixed to the back of the uterus at the uterosacral ligament level using four (ProTac) staples and four Vicryl (polyglactin 2-0) sutures for secure fixation (Figure 1D). The other end of the mesh was sutured above the sacral promontory by four ProTac staples then the mesh was covered with the peritoneum by Vicryl 2-0 sutures. Operative time was 60 minutes; histopathology confirmed benign leiomyoma.

The procedure went very well and the patient was discharged home after a couple of days. A follow-up examination after 6 months revealed a well-supported uterus and vaginal walls (stage I modified Baden and Walker) with good patient satisfaction. The patient did not have any new pelvic floor or urinary symptoms.

## Discussion

Uterine prolapse is the herniation of the uterus into or beyond the vagina as a result of failure of the ligamentous and fascial supports. It often coexists with prolapse of the vaginal walls, involving the bladder or rectum. In the UK, the disorder accounts for 20% of women waiting for major gynaecological surgery.

Uterine prolapse may present for years without troublesome symptoms and is often found incidentally during vaginal examination for other reasons. On the other hand, it can be extremely symptomatic and affect quality of life. Symptoms include increased vaginal discharge, a dragging sensation in the lower abdomen and back, a feeling of vaginal fullness and also a reduction in symptoms when lying down. With severe prolapse, when the uterus is bulging out of the vagina (procedentia), the skin may become irritated, raw and infected.

Uterine prolapse is associated with anterior or posterior vaginal wall prolapse (cystocoele and rectocele) in the majority of cases. Therefore, it may cause altered frequency of micturition, urgency or stress incontinence in associated cystocoele or difficulty emptying the bowel, a feeling of rectal fullness and sexual dysfunction in cases of rectocele.

Surgical treatment of female genital prolapse is a common procedure, but evidence for the most appropriate method of surgical repair is lacking. This is probably due to several aspects such as the relatively weak correlations between the symptoms of the prolapse and the size and extent of the anatomical defects of the pelvic floor; the difficulty in defining patients with identical genital prolapse; and the difficulty in performing uniform surgery in these patients. Thus, randomised studies of surgical methods may be more difficult to conduct in prolapse surgery [1].

Nichols emphasised the importance of identifying all sites of pelvic weakness at the time of prolapse surgery so that all sites might be repaired at the same time, sparing the patient the expense, pain and inconvenience of future readmission for further surgery [2].

A variety of surgical treatments for uterine prolapse with variable success rates have been described in the literature. Historically, vaginal hysterectomy remains the accepted surgical treatment for women with uterine prolapse. However, vaginal hysterectomy alone fails to address the pathological cause of the uterine prolapse [1]. The prolapsed uterus is not a cause, it is a result. The Manchester repair has been used as an alternative for women wishing to avoid hysterectomy. However, this operation has a high failure rate, associated with obstetrics complications and may cause difficulty sampling the cervix and uterus in the future.

As the laparoscopic technique advances along with understanding of the anatomy and physiology of pelvic support, the importance of site-specific repairs and post-hysterectomy support is being recognised.

For women with uterine prolapse wishing uterine preservation, laparoscopic suture hysteropexy is an advocated procedure [3]. In this procedure, the pouch of Douglas is closed and the uterosacral ligaments are plicated and reattached to the cervix. Forty-three women with symptomatic uterine prolapse were prospectively evaluated and underwent laparoscopic suture hysteropexy with a mean follow-up of 12 ± 7 months. On review, 35 women (81%) had no symptoms of prolapse and 34 (79%) had no objective evidence of uterine prolapse. The authors concluded that laparoscopic suture hysteropexy is effective and safe in the management of symptomatic uterine prolapse. The result is physiologically correct, without disfiguring the cervix.

An alternative approach to this problem is sacrohysteropexy. This involves suspending the uterus at the level of the uterosacral ligament to the sacral promontory using a non-absorbable mesh. By using a laparoscopic approach, we combined a more physiological and anatomical treatment of the prolapse together with quicker recovery. From our own experience, this suspension will correct mild cases of anterior vaginal wall prolapse (cystocoele) by further elevation of the vaginal axis. The potential advantages of a laparoscopic approach include quicker recovery and a reduction in adhesion formation, which is beneficial in women wishing to preserve their fertility. Ureteric injury is minimised compared with the vaginal approach as the ureters can be directly visualised [4]. Complications are rare and include retroperitoneal haematoma and large bowel injury during peritoneal dissection over the sacral promontory, at 2% each. Late complications include mesh erosion and recurrence of prolapse.

Uterine fibroid is a rare cause of uterine prolapse as only in large pedunculated fibroid, polyps may be prolapsed or cause uterine inversion. In our patient, we believe that the uterine fibroid was an innocent feature but its posterior position made mesh positioning impossible. Laparoscopic removal of the myoma is thought to have the dual advantages of making mesh positioning accessible and reducing uterine weight and the effect of gravity.

Recently, total surgical kits (new generation meshes including Prolift, Seratom and Avaulta) have been used successfully via the vaginal route for total prolapse preserving the uterus [5]. The kits also have several advantages such as short operation time, fewer complications and morbidity, easy application and a higher success rate. The occasional disadvantage is the erosion rate.

Reviewing the literature, the majority of the published studies so far have been on abdominal sacrohysteropexy. The largest review of 30 cases was by Barranger *et al*. [6]. The mean age of the women was 35.7 years (range 29-43 years). The women were all parous and had the Burch procedure and posterior colporrhaphy performed at the same time. Intra-operative and postoperative complications occurred in two patients (6.6%) and four patients (13.3%), respectively. Vaginal mesh erosion occurred in one woman. The mean objective and subjective follow-up periods were 44.5 months. Two cases of recurrent uterovaginal prolapse (6.6%) were described and three women had pregnancies that were conceived spontaneously.

The other follow-up studies were of 13 and 20 women, respectively [7,8]. No intra-operative or postoperative complications were reported. Recurrence of prolapse was minimal at 5% over 25 months' follow-up. The authors concluded that abdominal sacrohysteropexy is effective and safe in the treatment of uterovaginal prolapse in women who wish to retain their uteri. It maintains a durable anatomic restoration, normal vaginal axis and sexual function. The success rate is excellent for correcting prolapse, and the complications are minimal.

Recently, Rosenblatt *et al*. have published the largest case series of 40 women who underwent laparoscopic sacrocervicopexy [9]. Pelvic organ prolapse quantification system measurements were used and apical support was evaluated using point C. Mean C was -1.13 (+9 to -4) pre-operatively, -5.28 (-3 to -13) 6 weeks postoperatively, -5.26 (-3 to -8) 6 months postoperatively, and -4.84 (-3 to -7) 1 year postoperatively.

## Conclusion

We believe that, in the absence of indications for hysterectomy, laparoscopic sacrohysteropexy has an important role as a treatment for uterine prolapse. Correct mesh positioning plays an important factor in its long term success.

## Consent

Written informed consent was obtained from the patient for publication of this case report and any accompanying images. A copy of the written consent is available for review by the Editor-in-Chief of this journal.

## Competing interests

The authors declare that they have no competing interests.

## Authors' contributions

RF assisted in the operation, wrote the draft manuscript and literature review. JB was the primary surgeon, and reviewed and commented on manuscript
